# Innovative bioreactor designs for enhancing electron transfer, mass transfer, and product yields in microbial electrosynthesis

**DOI:** 10.3389/fbioe.2026.1888370

**Published:** 2026-08-24

**Authors:** Swabhiman Mohanty, Sriya Priyadarsini Dash, Rahul S. Tanpure, Samayita Chakraborty, Byong-Hun Jeon, Bikram Basak

**Affiliations:** 1 Department of Earth Resources and Environmental Engineering, Hanyang University, Seoul, Republic of Korea; 2 Centre for Research in Pure and Applied Sciences, Jain University, Bengaluru, India; 3 Center for Creative Convergence Education, Hanyang University, Seoul, Republic of Korea; 4 Petroleum and Mineral Research Institute, Hanyang University, Seoul, Republic of Korea

**Keywords:** biofuel, bioreactor designing, mass and electron transfer, microbial electrochemical system, organic waste valorization

## Abstract

Microbial electrosynthesis (MES), a form of bioelectrochemical system (BES) is an emerging versatile platform for the electrobioconversion of CO_2_ and diverse array of organic wastes into fuels and chemicals. However, its practical deployment suffers from certain drawbacks, including poor extracellular electron transfer (EET), mass transfer, high internal resistance, and product inhibition. Engineered bioreactor designs can help overcome these limitations. Novel bioreactor designs, such as zero-gap flow reactors, plate reactors, gas diffusion cathodes, porous flow-through, packed-bed electrodes, rotating membrane-less bioreactors, and electrodialysis-integrated devices, have improved current density, electron flux, hydrogen retention, ionic transport, Coulombic efficiency (CE), substrate utilization, and in situ product recovery. This mini review presents recent advances in the design of microbial electrosynthesis bioreactors, with an emphasis on the engineering strategies that enhance electron and mass transport, product titers, and volumetric productivity. Future progress in MES will rely on integrated reactor architectures and separation strategies for carbon-neutral biomanufacturing.

## 1 Introduction

Bioelectrochemical systems (BES) integrate microbial metabolism with electrode reactions to mediate extracellular electron transfer (EET), enabling energy recovery, pollutant removal, wastewater treatment and the production of value-added chemicals. Major BES configurations include microbial fuel cells (MFCs), which generate electricity via microbial oxidation of organic substrates; microbial electrolysis cells (MECs), yields hydrogen and other reduced products under applied voltage; and microbial electrosynthesis (MES) which uses cathodic electrons to drive microbial conversion of CO_2_ to methane, acetate, and long-chain products (Dattatraya Saratale et al., 2022; Marchetti et al., 2025; Zegers et al., 2025).

The transition of BES from H-type cells to pilot-scale systems highlights the pivotal role of reactor architecture, electrode spacing and hydrodynamics in determining performance and scalability. Within this broader framework, MES has gained increased attention because it allows selective CO_2_ conversion under temperate conditions, unlike abiotic electrochemical systems that use high overpotentials and expensive metal catalysts (Jourdin and Burdyny, 2021; Lekshmi et al., 2023; Zegers et al., 2025).

Despite its development, MES continues to face challenges in achieving the same industrial performance, owing to limitations in current density, volumetric productivity, electron transport, and microbial community assembly (Chen et al., 2020; Winkelhorst et al., 2023). Traditional MES systems have produced low current densities (typically <1 mA cm^–2^ and rarely exceeding ∼10–20 mA cm^–2^), due to constraints in electron transfer, gas-liquid mass transfer, ionic transport, hydrogen retention, biofilm development, and separator resistance that hinder electron flux to electroactive microbes (Bian et al., 2025; Kracke et al., 2021).

Reactor architecture is central to MES intensification, as it determines substrate utilization, interfacial electron flux, microenvironment stability, product yield, and downstream separability (Dessì et al., 2023). Advanced reactor designs, including zero-gap plate reactors shorten ionic pathways and reduce resistance, gas diffusion cathodes enhance CO_2_ availability, bubble columns and gas-lift systems increase H_2_ transfer, and electrodialysis integrated configurations can enhance gas delivery, mass transfer and product recovery (Bajracharya et al., 2022; Deutzmann et al., 2025; Pan et al., 2025), yet suffer from challenges such as hydrodynamic complexities, membrane fouling, ionic losses and wetting of gas diffusion electrodes (GDEs) (Giddings et al., 2015; Stoller et al., 2019).

Performance assessment across MES reactors remains constrained by diverse and complicated benchmarking metrics including internal surface area of 3D electrodes, volumetric productivity, areal current densities and energy efficiency, sometimes without reporting cell voltage. A robust benchmarking framework should therefore include current density, CE, volumetric productivity, cell voltage, and the normalization basis used for key metrics (Jadhav et al., 2022; Jourdin and Burdyny, 2021). Technoeconomic assessments (TEA) further indicate that commercial feasibility of MES depends on balancing high performance and product titers with low capital and operating costs (Peralta et al., 2026).

This review focuses on recent developments in MES reactor design, highlighting reactor configurations and electrode geometries that enhance electron transfer, mass transfer, and product formation, rather than on MES microbiology in isolation.

## 2 Electron transfer and mass transfer bottlenecks in MES

The MES performance depends on electron and mass transfer. Two principal electron delivery pathways govern MES: direct electron transfer (DET) from the cathode to biofilms and H_2_-mediated transfer via electrochemically generated hydrogen. The DET is limited by biofilm conductivity and kinetic constants related to electron uptake. H_2_-mediated routes have become favorable in high-rate reactors because high electron fluxes can occur without thick and highly conductive biofilms. However, the advantages of these routes are offset by their increased dependence on H_2_ mass transfer into the reactor (Jourdin and Burdyny, 2021; Ying et al., 2024).

Optimal mass transfer gives further control over the MES. The limiting factors for mass transfer are physical and chemical gradients in the biocathode, e.g., gases and ions. The low solubility of H_2_ and CO_2_, the gas residence time, separator resistance, bubble formation and limited ion transport affect substrate availability and mass transfer (Bian et al., 2025). These microenvironmental gradients increase the overpotential and inhibit essential microbial processes, such as acetogenesis and methanogenesis. Consequently, increasing the electrode surface area does not increase the efficiency because the active surface area for microbial interactions decreases (Bian et al., 2025). The low solubility of H_2_ (∼0.75 mM) restricts the diffusive flux to <1 mA cm^–2^ under typical boundary layers before bubble formation. Without improved gas delivery or retention, the current densities remain significantly below the theoretical limits, rendering them insignificant (Kracke et al., 2021). These bottlenecks explain why MES intensification requires the coordinated enhancement of interfacial electron flux, gas transfer, and ionic transport, rather than surface area enlargement.

## 3 State-of-the-art reactor architectures for MES intensification

**FIGURE 1:**
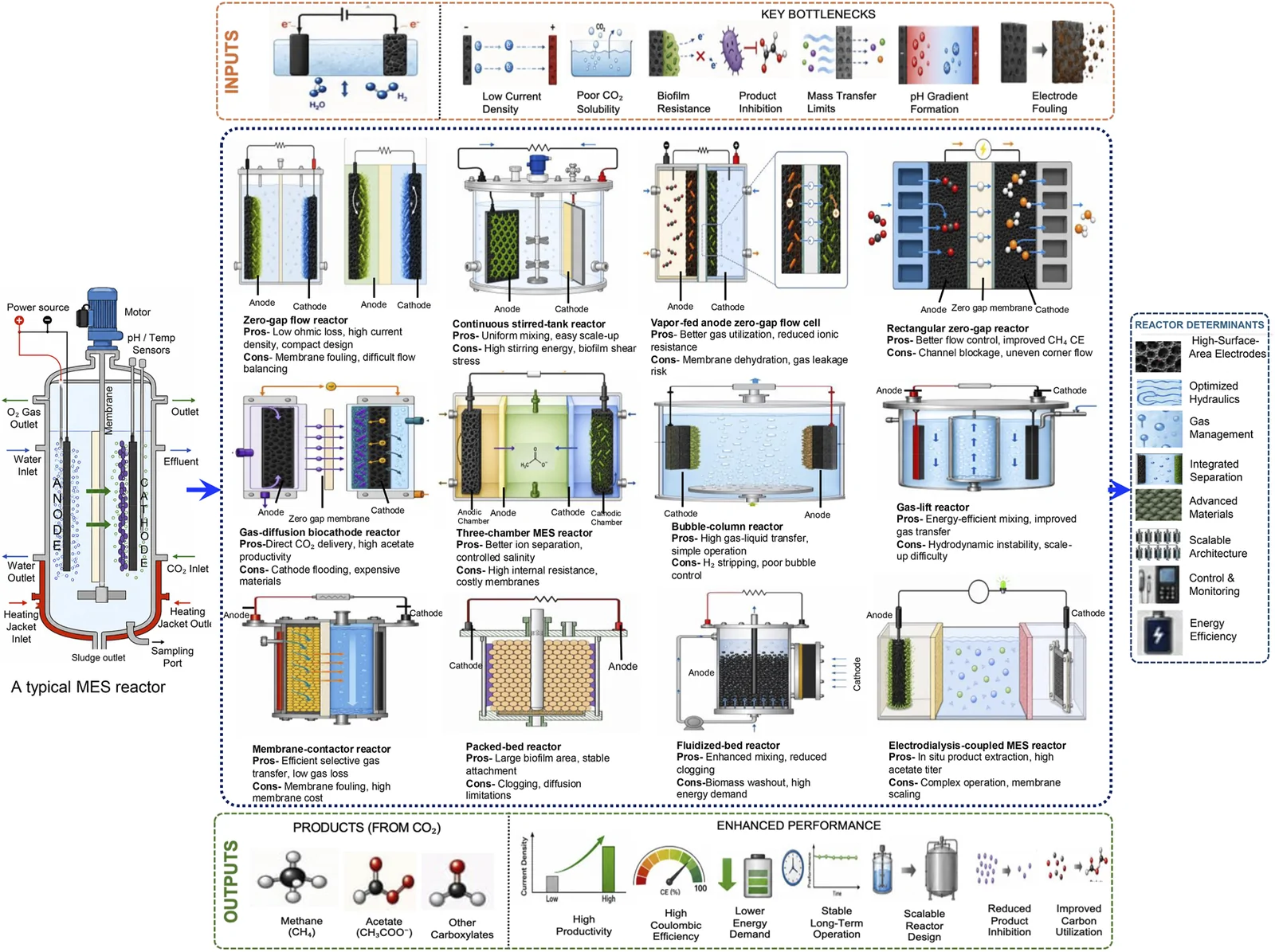
Engineered Reactor designing for Microbial Electrosynthesis from CO_2_. This schematic provides an overview of the reactor engineered for microbial electrosynthesis (MES), wherein reactor types and configurations are mapped against the key physicochemical and biological considerations that influence system performance. A common reactor configuration for MES along with the major limitations are highlighted that include limited current input, low CO_2_ supply, diffusion limitation in biofilm formation, product inhibition, mass transfer limitation, pH gradients, and electrode fouling. A comparison is made between representative reactor configurations in relation to the limitations associated with their design. The various design considerations for developing future generation MES systems are presented as well, such as electrode materials, reactor hydraulics, separation, process monitoring, and energy efficiency among others.

MES reactor designs are intended to improve efficiency by mitigating electron transfer and mass transfer. However, enhancement in one area often come at expense of stability, operability or scalability. Thus, a comparison between the different reactors is necessary. Recent high-performance MES systems have been classified into mechanistically distinct reactor classes (Table 1) (Figure 1).

**TABLE 1:** Summary of reactor designs and representative performance metrics (normalization basis).

Reactor configuration	Mechanism	Representative performance	Advantages	Disadvantages	Key takeaways	References
Zero-gap plate reactor with Ni-Mo-plated porous reticulated vitreous carbon (RVC) flow-through cathode	- Reduced internal resistance - High-surface-area flow-through cathode -Enhanced H_2_ mass transfer and electron delivery	- Methane >10.8 L L^-1^ d^-1^ -Acetate 3.5 g L^-1^ h^-1^ - Acetate titer 14.3 g L^-1^ - Coulombic efficiency (CE) up to 90% -Energy efficiency up to 30%	- High methane and acetate productivity - Superior electron donor utilization - Compact and modular design - Performance approaching gas and glucose fermentation	- Productivities still below commercial-scale gas fermentation; gas crossover -Diffusive losses across membranes -Optimization of electrode porosity and flow distribution -Clogging control -Reactor reproducibility required for scale-up	-Low resistance - High current -Among the highest productivity MES systems reported	(Deutzmann et al., 2025)
Zero-gap flow cell with vapor-fed anode	- Zero-gap electrode spacing - Low ohmic resistance - Vapor-fed anode minimizes pH gradients -Flow-through carbon felt cathode enhances mass transfer	- Methane 2.9 ± 1.2 L L^-1^ d^-1^ -Current density 17.4 A m^-2^ -Acetate 940 mmol m^-2^ d^-1^ - Ohmic resistance 2.4 ± 0.5 mΩ m^2^	- Catholyte maintained near neutral pH - low internal resistance - Improved methane and acetate production - Energy-efficient operation	- Methane productivity still below industrial requirements - Dependence on noble-metal catalyst -Requires gas management	-Enhanced gas delivery - Strong process intensification potential	(Baek et al., 2022)
Three-chamber MES with gas-diffusion biocathode (3D-printed)	- Improves CO_2_ delivery - Moderate salinity increases conductivity - Reduces ohmic losses	- Acetate 55.4 g m^-2^ d^-1^ (0.89 g L^-1^ d^-1^) - CE 82.4%	- Improved CO_2_ mass transfer -Modular stackable design, -Scalable architecture -Reduced power demand	- Acetogenic activity inhibited above ∼6 g L^-1^ -Production rate still below industrial benchmarks -Salt precipitation	- High area - Complex control - Improves CO_2_ utilization - Supports modular reactor designs	(Dessì et al., 2023)
Electrolytic gas-lift (draft-tube bubble column) reactor	- Internal draft tube improves gas-liquid circulation - H_2_ mass transfer, and mixing efficiency	- Current density 337 A m^-2^ -Acetate titer 20.5 g L^-1^ - Volumetric mass transfer increased 76.6%	- Excellent mixing - High current density -Strong scale-up potential	- Performance remains dependent on efficient H_2_ transfer -Challenge in hydrodynamic control during scale-up	- Improved mixing - Gas transfer - Higher scale-up potential	(Pan et al., 2024)
Electrolytic bubble-column reactor with external hollow-fiber gas–liquid contactor	- Extended H_2_ retention -Gas recirculation -Recovery of unused gases -Enhanced gas–liquid mass transfer	- Acetate 1.15 g L^-1^ d^-1^ -Acetate titer 34.5 g L^-1^ - CE 64% average	- Large reactor volume (5.5 L) -High acetate titer -Efficient gas utilization	- CE declines under high-current operation; reactor -Complexity increased by gas-recycling loop	- Enhanced mass transfer -Gas utilization efficiency	(Cui et al., 2023)
Packed–fluidized bed cathode MES with granular activated carbon (GAC)	-Increases surface area, CO_2_ adsorption, microbial attachment - Mass transfer; direct CO_2_ delivery through packed bed	- Acetate production rate 0.16 g L^-1^ d^-1^ -Current density 0.47 mA cm^-2^ initially - CE up to 55% -Butyrate 0.66 g L^-1^	- Low-cost cathode, improved CO_2_ bioavailability - Enhanced biofilm growth -Higher productivity than non-packed and bare-GAC control	- low productivity compared with advanced MES systems -Potential clogging requires periodic fluidization - CE remains moderate	-High surface area - Strong transport	(Shakeel and Khan, 2022)
Fixed/fluidized granular activated carbon bed cathode reactor	-Increases cathode surface area and biofilm attachment	- Fixed bed acetate 204 ± 2 mg L^-1^ d^-1^ - Current density nearly 2× higher than fluidized bed	- Simple architecture -High surface area - Fixed bed outperformed fluidized configuration	- Reduces charge transfer efficiency and acetate productivity -CO_2_ transfer remained limiting	-Scalable - Transport limited -Low-cost electrode architecture with enhanced microbial attachment	(Vassilev et al., 2024)
Membrane-less rotating-disc bioelectrochemical reactor (RDBER)	- Improve mass transfer and provide large scalable electrode area (up to 1 m^2^)	−87% cathode coverage after 24 days - Anodic current density 130 μA cm^-2^ - H_2_ production 0.43 L L^-1^ d^-1^	- Scalable membrane-less design - large electrode surface area, suitable for anodic and cathodic biofilms	- Biofilm distribution heterogeneity -Mass-transfer limitations at larger scales require optimization	-Compact mixing -Enhanced design -Reduces reactor complexity	(Hackbarth et al., 2023)
Electrodialysis-coupled electrolytic bubble-column MES	- In-situ acetate extraction -Recovers product inhibition and improves gas utilization	- Acetate titer 98.1 g L^-1^ -CE 84% -Gas uptake efficiency 100%	- Very high acetate titer - reduced product inhibition −98.7% lower NaOH consumption -Strong industrial relevance	- Additional electrodialysis unit increases system complexity and production cost	-High titers - Integrated separation -Operational complexity	(Pan et al., 2025)
Electrochemical continuous stirred-tank reactor (E-CSTR) with tubular electrochemical cell	- In-situ H_2_ generation within a stirred tank - Enhanced gas–liquid mixing - Prolonged H_2_ retention - Suspended-cell H_2_-mediated electron transfer	- Methane 0.53 L L^-1^ d^-1^ -Current density 119.9 A m^-2^ - CE 98.1% -Energy efficiency 20%	- Large-scale operation (20 L), excellent robustness -Electron recovery -Uniform mixing -scalable industrial reactor architecture	- Oxygen crossover through membrane at high current densities inhibited methanogenesis -High stirring/heating energy demand - Volumetric productivity still below compact high-rate MES reactors	-Flexible -Continuous operation -Compatible with established industrial bioprocessing infrastructure	(Shang et al., 2023)

CE, coulombic efficiency; GAC, granular activated carbon; RVC, reticulated vitreous carbon.

### 3.1 Zero-gap and compact plate reactors

Compact zero-gap plates and flow reactors reduce internal resistance and facilitate uniform ionic pathways by bringing electrodes and separators into proximity, thereby enabling high methane and acetate productivities (Baek et al., 2022; Deutzmann et al., 2025). Recently, Ni–Mo-plated porous cathodes in a compact zero-gap plate reactor achieved methane and acetate production rates of >10 L L^−1^ d^−1^ and 3.5 g L^−1^ h^−1^, respectively, establishing MES scalability at industrial benchmarks (Deutzmann et al., 2025). However, these systems are also accompanied by changes in local pH gradients, and ion accumulation near membrane interfaces creating scale-up and operational challenges with complex feeds (Deutzmann et al., 2025; Fathima et al., 2024; Jourdin and Burdyny, 2021). Moreover, porous cathode architecture coupled with optimized flow distributions can reduce dead zones, facilitating (Bian et al., 2025).

### 3.2 Gas-diffusion and gas-fed systems

Gas diffusion cathodes address the low solubility of CO_2_ by supplying the substrate directly to the catalyst biofilm interface circumventing the limitations associated with the mass transfer and substrate availability. Three-chamber configurations with gas diffusion biocathodes have enhanced CO_2_ delivery and facilitated acetate production of 55.4 g m^−1^ d^−1^ (0.89 g L^−1^ d^−1^) at 82.4% CE, while controlling conductivity and salinity (Dessì et al., 2023). Although, gas-diffusion cathode increases the supply of the substrate, its sustained efficiency can be limited by flooding, wetting, salt precipitation, and maintenance issues that have been generally neglected during productivity-based studies (Dessì et al., 2023; Jourdin and Burdyny, 2021).

### 3.3 Bubble-column, packed-bed, and fluidized-bed systems

Bubble-column, gas-lift, and membrane contactor reactors intensify H_2_-mediated MES by enhancing gas-liquid transfer (kLa) and H_2_ retention, resulting in high current densities (up to 337 A m^−2^) and increased acetate production compared to other conventional reactors (Pan et al., 2024). Even with high quality gas transfer, the reactors may be prone to limited liquid circulation, longer mixing rates which can produce pH and temperature gradients that affects process uniformity (Bokelmann et al., 2025). Packed and fluidized-bed reactors offer large surface areas for mass transfer, while fixed-bed electrodes can generate higher current densities and acetate yield. Furthermore, enhanced mixing in fluidized systems can lower charge efficiency (Miehle et al., 2026; Shakeel and Khan, 2022; Vassilev et al., 2024). In contrast to their benefits, the process is greatly affected by pore clogging, biomass accumulation, biofilm scouring, and particle attrition, reflecting the inherent trade-offs between process intensification and reactor durability (Dhiman et al., 2026; Fathima et al., 2024; Sankaran et al., 2026).

### 3.4 Rotating, membrane-less systems and electrodialysis-integrated MES reactors

Rotating reactors increase mass transfer, by thinning boundary layer and elevating shear induced biofilm, which can mitigate clogging and electrode stability, conversely electrode discs are affected by angular at the outer edge where hydrodynamics conditions differ (Hackbarth et al., 2023). Membrane-less MES systems reduce the membrane costs and eliminate the risk of membrane fouling but demands flow control to maintain redox reactions (Georgiou et al., 2022). Electrodialysis coupled MES delivered productivity of 4.32 g L^−1^ d^−1^ acetate at a titer of 98.1 g L^−1^ while reducing NaOH consumption per unit acetate by 98.7%, underscoring the potential of integrated separation for in situ product recovery, alleviation of product inhibition and reduced. Downstream costs (Pan et al., 2025). Nevertheless, integrated separation increases the capital expenditure and may introduce additional fouling and energy penalties (Pan et al., 2025).

Overall, future MES scale-up would revolve around achieving high current densities, while maintaining electron and mass transfer and long-term operational stability.

## 4 Strategies to enhance electron transfer

Electron transfer intensification begins at the cathodes. The cathode performance is regulated by the electrode microstructure, interfacial chemistry, and microbial physiology. Three-dimensional (3D) conductive porous electrodes increase the active surface area, with flow patterns and gas disengagement supporting microbial attachment via microstructures (Erbay et al., 2015). Functionalization of cathodes with metal oxides, layered double oxides, or conductive carbon frameworks reduces resistance and enables direct electroautotrophic selection. Bimetallic Fe–Mn oxide coating improved acetate production from 28 to 78 g m^−2^ d^−1^ and enriched homoacetogens (Du et al., 2024), while Ni–Fe-layered double-oxide/carbon nanotube hybrid cathodes improved acetate production by lowering resistance. This suggests that catalyst–microbe compatibility aids electron transfer and overall performance (Chen et al., 2026).

Recent studies using 3D-printed graphene aerogel/polypyrrole biocathodes reported methane production of 1,672 ± 131 mmol m^−2^ d^−1^, surpassing that of carbon felt electrodes (He et al., 2023). Rotating and mechanically refreshed electrode designs are alternative strategies that alleviate diffusion layer restrictions and encourage biofilm renewal while preventing clogging (Hackbarth et al., 2023). Biofilm localization strategies, such as bioprinted or immobilized biocatalyst layers, shorten the start-up duration and position dense biomass closer to the electron source (Read et al., 2010). Synthetic biofilm strategies, including the 3D bioprinting of cell-laden hydrogels directly onto cathodes, improve acetate productivity by reducing startup limitations and localizing dense electroautotrophs at the cathode surface (Krige et al., 2021). Together, these cathode enhancements and reactor designs can improve the residence time, gas distribution, and ionic conductivity for maximum output, while the next challenge lies in enhancing mass transfer.

## 5 Strategies to enhance mass transfer

In modern MES platforms, mass transfer enhancement is achieved through refined gas delivery and the control of ionic microenvironments. H_2_-mediated systems depend on the optimization of bubble size, headspace gas loss, recirculation patterns, residence time distribution, and H_2_ supply equilibrium (Kracke et al., 2021; Liu et al., 2023). Rectangular porous electrodes with flow configurations and engineered inlet geometries can improve retention and reduce dead zones, whereas gas-diffusion cathodes offer an alternative by delivering gas directly to the catalytic interface, avoiding catholyte saturation with a mass transfer load (Bian et al., 2025; van der Lee et al., 2026). Dessì et *al*. demonstrated that a gas-diffusion MES reactor can achieve acetate formation rates of 55.4 g m^−2^ d^−1^ at applied current densities ranging from 0.25 to 1.0 mA cm^−2^ (Dessì et al., 2023). Hydrodynamics and selectivity cannot be optimized in isolation. Enhanced circulation increases the current density; however, it decreases hydrogen retention, lowering the CE of methane or acetate due to hydrogen loss. The choice of separator, temperature, and electrolyte conductivity requires the integration of product removal into the mass-transfer design (Bian et al., 2025).

## 6 Reported product yields and benchmarking metrics

Benchmarking MES performance can often be misleading because reported results are heavily influenced by the normalization choices, electrochemical conditions, and product yield. Metrics such as titer, productivity, current density, and (CE) are broadly used; however, TEA consider current density, energy efficiency, and cell voltage closely aligned with practical feasibility (Puig et al., 2021; Renju and Singh, 2026). In MES, selection of normalization parameters determine performance interpretation. Based on the results, whether reported per the projected area, internal surface, geometry, electrode volume, or catholyte volume, the same system may appear either highly efficient or relatively weak. This variability highlights that choosing a denominator is complex but fundamentally influences the benchmarking results (Jourdin and Burdyny, 2021). Despite the frequent use of CE to measure the effectiveness of MES, a high CE does not always signify efficient reactor performance, since productivity, energy efficiency, and scale-up remains limited. Hence, CE must be evaluated together with current density, productivity, and energy utilization (Jadhav et al., 2022). Similarly, the highest product titer is not a direct indicator of superior performance, rather such productivities need to be considered with energy requirements, membrane costs, separation complexity, reactor intensification and practical operability (Alrabiah et al., 2026; Jourdin and Burdyny, 2021).

New studies have found that biocatalytic biofilm reactors and zero-gap reactors generate higher current densities by reducing ohmic resistance and promoting better gas transport (Bian et al., 2025). In comparison, MES coupled with electrodialysis has resulted in the most efficient production of acetate, with titers of up to 98.1 g L^−1^ on average at 4.32 g L^−1^ d^−1^, demonstrating greater efficiency in inhibition management and reduced use of chemicals (Pan et al., 2025). However, these systems suffer from membrane fouling, Cathode flooding and increased maintenance that are rarely reflected under conventional benchmarking metrics (Fathima et al., 2024). Engineering details such as the reactor dimensions, inter-electrode distance, membrane design, internal resistance, gas circulation method, and operating conditions should also be stated along with other measurements since they influence mass transport, ohmic effects, and scale-up possibilities (Jadhav et al., 2022; Singhal et al., 2026; Zhu et al., 2026).

Robust benchmarking requires multidimensional assessment rather than overdependence on individual metrics. For cross-study comparison, the studies should include a framework that includes areal and volumetric current densities, product titer, volumetric productivity, CE, Faradaic efficiency, cell voltage, electrode potential, catalyst loading, reactor configuration, inter-electrode distance, and internal resistance (Dutta et al., 2024; Singhal et al., 2026). Moreover, methods of digitalization like SCFCM (Sequence-constrained Fuzzy c-means) have found that operational parameters can be utilized for identifying product-selective phases and for optimization of the processes (Albarracin-Arias et al., 2026). These parameters critically influence mass transfer, ohmic loss, power requirements, and scale-up capability of the process. The absence of these parameters can make a reactor appear excellent at one performance metric, yet fail to be industrially feasible.

## 7 Techno-economic and scale-up considerations

Despite considerable advancements in reactor efficiency, MES is still limited by electricity demand, materials costs, and scaling up challenges. The techno-economic study highlights electricity as a major cost driver, which largely depends on the current density and cell voltage. For instance, gas-lift and zero gap reactors achieved current densities higher than 100–300 A m^-2^, although preserving high energy efficiency increases cost with ohmic and mass transfer limitations (Pan et al., 2024). It is therefore necessary to design reactors with reduced internal resistance through electrode spacing, conductive electrolytes and improved hydrodynamics (Jourdin et al., 2020; Tian et al., 2023).

Beyond electricity demand, fouling of the membrane, degradation, gas crossover, pH gradients, and separator replacement all increase operating expenditures. Thus, material and design selection becomes crucial: compact, modular, and membrane-less design, low-cost electrode materials reduce maintenance, but compromises with selectivity, control, and stability (Georgiou et al., 2022; Pan et al., 2025; Tian et al., 2023)At larger scales, the role of gas management becomes critical, particularly for MES driven by H_2_ requires effective gas-liquid transfer to sustain high CE. In this context, preparation of a CoP nanowire-coated nickel foam superaerophobic cathode, minimized H_2_ bubble size from approximately ∼300 to ∼100 μm, increased the mass-transfer coefficient by 129% (KLa = 0.32 min^-1^), and methane production to 2.31 L L^-1^ d^-1^ at 166.7 A m^-2^ while maintaining CE above 80% (Gao et al., 2025). MES combined with electrodialysis also achieved acetate titers close to 100 g L^-1^ while saving chemicals up to 99%, demonstrating the contribution of integrated separation technology overcoming product inhibition (Pan et al., 2025).

From a scale-up perspective, reactor modularity is increasingly viewed as a more practical alternative than simple geometric enlargement. Modular systems enable maintenance without complete process shutdown, improve operational flexibility, and reduce risks associated with uneven current distribution, mass-transfer limitations, and hydrodynamic heterogeneity (Barman et al., 2026; Feng et al., 2026; Pan et al., 2025; Shang et al., 2023). The integration of computational fluid dynamics (CFD) and AI/ML-based surrogate modeling methods like deep neural networks (DNNs) represents a powerful framework for understanding and optimizing MES reactor design and scale-up. Analysis of flow distribution, transport of gases and liquids, as well as the local mass transfer limitations are beyond experimental resolution, in such cases these approaches provide in-depth insights regarding the hydrodynamic constraints affecting reactor operation. These tools are well suited for designing compact reactors with zero-gap or gas-fed reactors, where the bubble behavior and concentration gradients strongly influence current density and energy efficiency of production. In this context, CFD modeling facilitates the rational optimization of electrode spacing, channel geometry, electrolyte flow and gas management; while surrogate modeling/DNNs can predict performance of the system under a wide range of conditions (Dhakane and Yadav, 2024). However, long-term operation still faces challenges related to membrane fouling, electrode degradation, biofilm overgrowth, catalyst deactivation, and gas crossover.

## 8 Discussion

Contemporary studies on MES indicate that the reactor architecture, rather than the cathode material alone, largely determines the high current densities for useful productivity (Deutzmann et al., 2025). Reactors such as zero-gap plate reactors, gas-diffusion cells, gas-lift or bubble-column systems, and integrated separation reactors are effective. They increase electron flow, reduce ohmic losses, refine gas delivery, and stabilize the cathode microenvironment (Baek et al., 2022; Cui et al., 2023; Dessì et al., 2023). In this regard, electron-transfer pathways have major design implications. DET-based systems require close cathode-biofilm contact. Therefore, favored by designs with large surface area, porous or zero-gap structures where electrons can be transferred directly at the interface, nonetheless, such systems might be prone to biofilm aging, cathode fouling, and low electrode surface utilization efficiency problems. On the other hand, H_2_ systems depend on efficient production, retention, and consumption of hydrogen, which makes gas-augmented designs like bubble column, gas lift, and gas diffusion reactors more appropriate, but which are restricted by gas transfer losses and incomplete hydrogen utilization (Cai et al., 2024; Gao et al., 2025; Liu et al., 2023).

Advances in MES have established new performance metrics, with systems attaining high CEs (80%–90%) and production rates nearing those of conventional fermentation processes (Cabau-Peinado et al., 2024; Deutzmann et al., 2025; Shang et al., 2023). However, these improvements have been achieved mainly under idealized conditions, such as controlled cultures, high-purity inputs, and engineered cathodes, which limit their immediate scalability. Thus, effective benchmarking treats systems as complete transport units rather than CE (%) alone (Jourdin et al., 2020). A high CE can coexist with limited volumetric productivity, whereas a high current density can be counterproductive at high cell voltages and low gas utilization rates (Deutzmann et al., 2025). Long-term operation is challenged by biofilm aging and cathode fouling whereas product inhibition and poorly characterized responses to intermittent power hinder progress (Marshall et al., 2013; Stoller et al., 2019). Overcoming these limitations requires a mechanistic understanding of EET integrated with electrochemical and multi-omics analyses, as well as a model-guided reactor design to validate the performance at the pilot scale. The most informative MES studies reported the real current density (A m^–2^), volumetric and areal productivity, product titer, CE (%), cell voltage or energy efficiency, reactor volume, and normalization of each metric (Chu et al., 2023; Jadhav et al., 2022). The integration of MES with separation systems and further development of TEA analysis models are vital for determining the feasible regimes of operation and scaling-up procedures. Regardless of many developments in this field, several barriers still prevent the practical application of MES in the industry.

## 9 Challenges and future directions

Three immediate challenges define the progress in this field. First, reports remain inconsistent, especially regarding the cathode area basis, operational volume, applied versus measured current density, and mass transfer coefficients (kLa), which limit benchmarking and cross-reactor evaluations (Jadhav et al., 2021; Jourdin and Burdyny, 2021). The second aspect is the long-term stability of the system operation under different power regimes. In intermittent operation, parameters like hydrogen consumption, selectivity, and biofilm activity take a long time to stabilize, requiring long validation tests. (Deutzmann et al., 2022). Third, barriers include the integrated scale-up and coordinated regulation of coupled factors, including salt dynamics, pH, and gas transport (Fathima et al., 2024).

Further advancements in MES will rely on co-engineering high performance reactors and separation technologies considering the constraints that remain in MES scaling downstream. The findings in electrodialysis-assisted acetate systems demonstrate that *in situ* extraction is effective at overcoming product inhibition (Martínez Sosa et al., 2026; Pan et al., 2025). More broadly, recent reviews have highlighted that integrating conversion and separation into one architecture through electrodialysis, membrane-driven electrolysis, or sorption-based systems is crucial for high-yield production and maintaining economically sustainable operations (Al Balushi et al., 2025).

## 10 Conclusion

MES has moved beyond the proof of concept. Reactor innovations that enable methane and carboxylate production at volumetric and areal rates have previously been considered unreachable. Low-resistance, compact, zero-gap configurations coupled with 3D cathodes and gas-fed configurations have eased the principal transport and electrochemical barriers, bringing MES closer to competing with established fermentation processes. Despite these advancements, gaps in understanding, stability, and integration remain. These critical gaps remain in terms of mechanistic understanding, operational stability, and processing integration. Future work should focus on standardized metrics, in-depth examination of microbe -electrode interactions, and implementation of stable, long-term, continuous operation. This will facilitate the transformation of MES into an operational system for sustainable chemical production.

Future advances in MES technology should focus on the standardization of performance metrics, deeper understanding of microbe-electrode interactions through sophisticated electrochemical and omics methodologies and the achievement of stable long-term continuous operations. Concurrent progress in process integration and scale-up strategies will be crucial in translating MES from laboratory into practical technologies for sustainable production.
